# Unlocking the promise of liquid biopsies in precision oncology

**DOI:** 10.1016/j.jlb.2024.100151

**Published:** 2024-03-21

**Authors:** Alejandra Pando-Caciano, Rakesh Trivedi, Jarne Pauwels, Joanna Nowakowska, Beatrice Cavina, Lovisa Falkman, Jessica Debattista, Szilárd-Krisztián Belényesi, Periyasamy Radhakrishnan, Mariano A. Molina

**Affiliations:** a Department of Cellular and Molecular Sciences, Universidad Peruana Cayetano Heredia, Av. Honorio Delgado 430, San Martín de Porres, Lima, 15102, Peru; b Subunit of Research and Technological Innovation, Instituto Nacional de Salud del Niño San Borja, Av. Javier Prado Este 3101, Lima, 15037, Peru; c Department of Cancer Biology, Mayo Clinic, Scottsdale, AZ, USA; d VIB-UGent Center for Medical Biotechnology, VIB, 9052, Ghent, Belgium; e Department of Biomolecular Medicine, Ghent University, 9052, Ghent, Belgium; f Molecular and Cell Biology Unit, Department of Pediatric Pulmonology, Allergy and Clinical Immunology, Poznan University of Medical Sciences, Poznan, Poland; g Doctoral School, Poznan University of Medical Sciences, Poznan, Poland; h Department of Medical and Surgical Sciences (DIMEC), Centro di Studio e Ricerca sulle Neoplasie (CSR) Ginecologiche, Alma Mater Studiorum-University of Bologna, 40138, Bologna, Italy; i Centre for Applied Biomedical Research (CRBA), University of Bologna, 40138, Bologna, Italy; j Department of Medical Sciences, Endocrine Tumor Biology, Uppsala University Hospital, Uppsala University, Uppsala, Sweden; k Pathology Department, Faculty of Medicine and Surgery, University of Malta, Malta; l School of Pharmacy and Pharmaceutical Sciences, Trinity College Dublin, Ireland; m Trinity Biomedical Sciences Institute, Trinity College Dublin, Ireland; n Trinity St. James’s Cancer Institute, Trinity College Dublin, Ireland; o Department of Medical Genetics, Kasturba Medical College, Manipal, Manipal Academy of Higher Education, Manipal, 576104, Karnataka, India; p Department of Pathology, Vrije Universiteit Amsterdam, Amsterdam UMC Location VUmc, Amsterdam, the Netherlands; q Cancer Centre Amsterdam, Imaging and Biomarkers, Amsterdam, the Netherlands; r Instituto de Ciencias Médicas, Las Tablas, Panama

**Keywords:** Liquid biopsy, Precision oncology, Cancer, Biomarker, Standardization, Clinical implementation

## Abstract

Liquid biopsies have emerged as a promising and minimally invasive alternative to traditional tissue biopsies for detecting and monitoring cancer. Liquid biopsies offer a comprehensive analysis of cancer genetics and tumor burden by examining circulating cells and cell-derived analytes using a variety of assays, including conventional PCR methods and cutting-edge tools like long-read sequencing and nanotechnology. However, there are still some limitations and challenges that need to be overcome for their implementation in clinical routine, including the need for further research on their sensitivity and specificity, cost-effectiveness, standardization, and regulatory approval. Despite these challenges, liquid biopsies have the potential to become widely used tools in oncology. Here we provide an overview of the current state of liquid biopsies, highlighting recent advancements in the field and their potential benefits in clinical settings for cancer patients. The article further discusses the challenges that need to be addressed in order to facilitate their application worldwide. Prompt resolution of these challenges can be achieved by fostering international research collaborations and establishing standardized guidelines for liquid biopsy sample management and studies.

## 1 Main text

**Fig 1:**
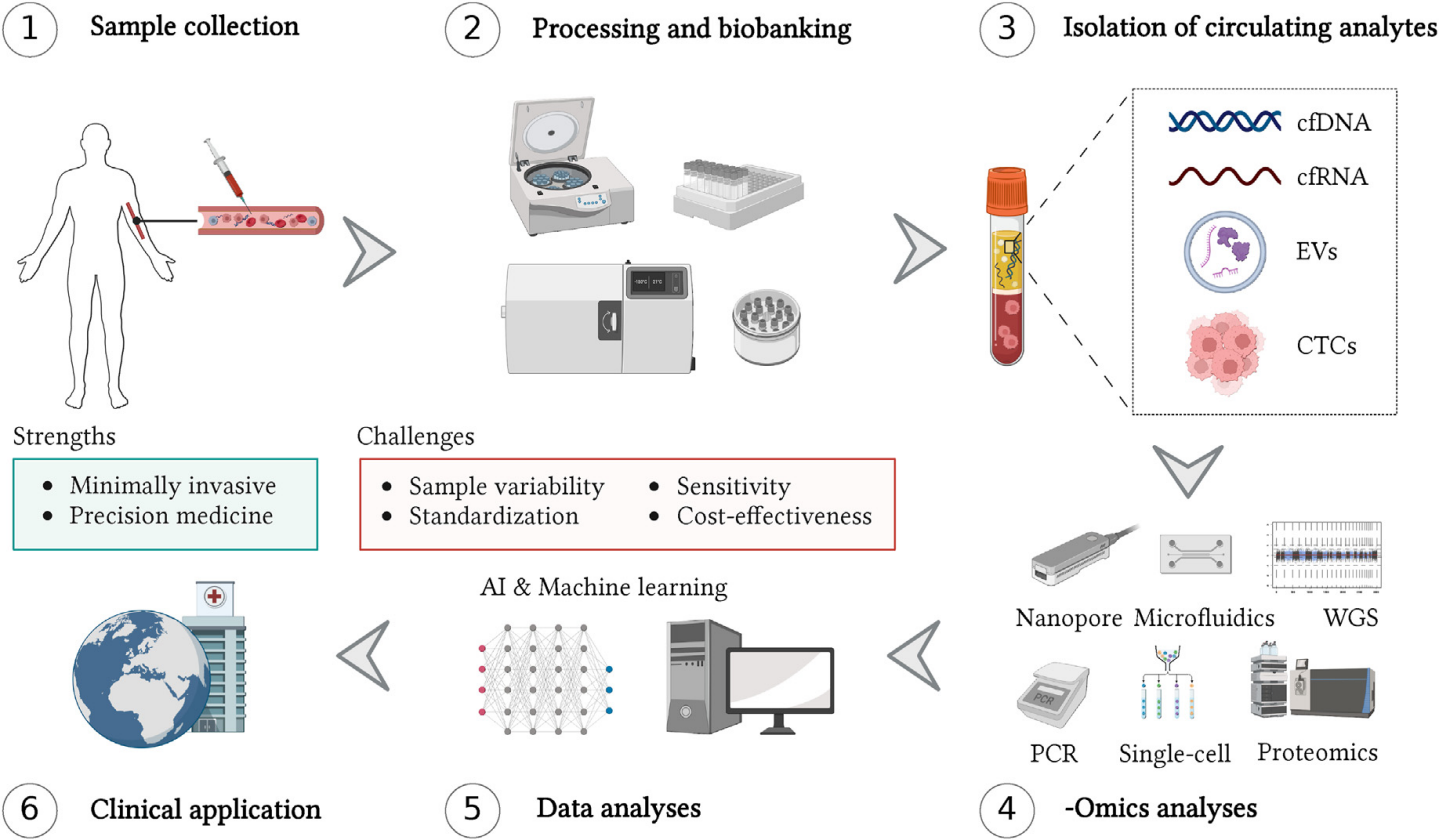
**Workflow of liquid biopsy in precision oncology**. This schematic illustration outlines the sequential steps involved in the liquid biopsy process for precision oncology. Starting with the collection of a liquid biopsy sample (e.g., blood), the workflow contains key stages, such as processing (e.g., centrifugation) and biobanking, followed by isolation of circulating analytes, including circulating tumor cells (CTCs), cell-free DNA (cfDNA), cell-free (cfRNA), and extracellular vesicles (EVs). Subsequent -omics analysis of these components allows for the identification of genetic alterations, such as mutations, copy number variations, and epigenetic modifications. The integration of comprehensive molecular profiling data through AI and machine learning algorithms aids in the characterization of the tumor's genomic landscape, guiding personalized treatment decisions and monitoring disease progression over time. An accurate liquid biopsy-assay for cancer detection, progression, or monitoring has the potential to be implemented in clinical routine worldwide.

Liquid biopsies represent a transformative approach in oncology and personalized medicine. They generally do not have the same limitations as tumor biopsy samples in terms of quantity and quality, the inability to assess intratumoral heterogeneities and the invasiveness of the collection procedure (Fig. 1). Liquid biopsies-based approaches can be applied worldwide, and they hold significant potential in low- and middle-income countries (LMICs), which have a considerably higher cancer-related mortality than high-income countries and lack the infrastructure for costly standard diagnostic procedures [1]. The recent advancements in evaluating diverse analytes and detection technologies [2,3] are remarkable. Nevertheless, the suboptimal reproducibility in liquid biopsy studies makes it necessary to evaluate this strategy and assess the recent developments in the field and the factors needing improvement to objectively translate liquid biopsy discoveries into results that directly benefit patients.

Next-generation sequencing (NGS) technologies have undeniably been crucial in advancing liquid biopsy research. Genome-wide sequencing [4], targeted sequencing [5], and long-read sequencing [2] offer high sensitivity and specificity for detecting copy number alterations (CNA) and mutations in a variety of analytes, such as circulating tumor cells (CTCs), circulating tumor DNA (ctDNA), and cell-free DNA (cfDNA) (Fig. 1) [6]. Yet, even though sequencing-based liquid biopsy assays exhibit high sensitivity, the trade between sensitivity and cost is a major concern in clinical practice. Further development of these sequencing techniques and user-friendly bioinformatics tools for analyzing NGS data remain essential for continued enhancement and evaluation of their precision, feasibility of routine implementation, and cost-effectiveness in oncology.

Single-cell analyses and PCR-based approaches have also emerged as indispensable tools for deciphering the genetic, epigenetic, and transcriptional diversity of cancer in liquid biopsies (Fig. 1). By employing single-cell methods, it is possible to examine the molecular profiles and clonality of cancer cells, uncovering rare subpopulations and dynamic changes that may go unnoticed with bulk analyses [7]. The detailed understanding of tumor heterogeneity and microenvironment, facilitated by these approaches, is key for developing precise treatment strategies in oncology. Furthermore. implementing high-sensitivity detection methods, such as digital PCR [8], can potentially uncover low-frequency DNA mutations and small non-coding RNA molecules in different body fluids that may otherwise go undetected. As the field of liquid biopsies progresses, researching the potential of these techniques could improve the translation of basic and clinical science into medical practice.

Another optimistic step in the field is the increasing discovery of sources for tumor markers, such as circulating extracellular vesicles (EVs), proteomes, and epigenomes (Fig. 1). EVs carry cellular materials (e.g., small RNAs, DNA, lipids, metabolites, and proteins) recognized as promising cancer biomarkers in liquid biopsies. The challenge lies in distinguishing reliable biomarker signatures from the vast pool of small molecules and proteins encapsulated and associated with EVs. Future EV-based studies should therefore adhere to the Minimal Information for Studies of Extracellular Vesicles (MISEV) guidelines [9] to increase rigor and reproducibility in EV research and employ the EV-TRACK knowledgebase [9], which records experimental parameters of EV-related studies. Moreover, the comprehensive study of the abundance, modifications, and interactions of proteins through proteomic analyses can make possible the identification of specific protein signatures that can serve as biomarkers for cancer detection, progression monitoring, and treatment response assessment [10,11]. Liquid biopsy protein profiling is gaining progressively more momentum with the advent of sensitive LC-MS/MS instruments operating in high-throughput that improve the robustness and reproducibility of data acquisition [12]. Likewise, epigenetic alterations, such as DNA methylation patterns and histone modifications, play a crucial role in cancer development and progression [13,14]. These epigenetic changes can be captured in liquid biopsies, providing valuable insights into the regulation of gene expression associated with tumorigenesis. Thus, the future of biomarker discovery and cancer-specific assay development in liquid biopsies will rely on sensitive detection methods and the possibility of incorporating multi-omics and multi-modal approaches.

Nanotechnology-based techniques also represent a promising approach for isolating and measuring circulating biomarkers in liquid biopsies [15]. These analytical techniques use nanomaterials, such as gold and magnetic nanoparticles, conjugated with antibodies and nucleic acid probes, liposomes, exosome-mimicking nanoparticles, and quantum dots, which can be modified on their surfaces to detect multiple biomarkers in a single sample, making the procedure time- and cost-efficient [16]. The development of microfluidic platforms like lab-on-a-chip systems has enabled highly efficient processing and analysis of liquid biopsy samples (Fig. 1). These systems are ideal for point-of-care testing, especially in LMICs [17]. These techniques allow for the detection and quantification of low levels of circulating biomarkers in body fluids in a more precise manner than conventional techniques. Similarly, nanotechnology-based assays can further enhance the stability of biomarkers found in liquid biopsies. Hence, the prospect of liquid biopsy-based diagnostics lies in the continued development and optimization of miniaturized, high-throughput, and point-of-care tests for improved cancer diagnosis and follow-up.

As the landscape of liquid biopsy research continues to expand, advancements in artificial intelligence (AI) and machine learning (ML) are increasingly being harnessed to enhance the interpretation and clinical utility of liquid biopsy data [18]. The sheer volume and complexity of information generated by liquid biopsies require sophisticated computational approaches for accurate analysis and interpretation. AI and ML algorithms can identify subtle patterns, predict disease progression, and aid in the identification of potential therapeutic targets [19,20]. This integration of cutting-edge technologies not only enhances the accuracy of liquid biopsy results but also holds the promise of developing predictive models that could revolutionize early cancer detection and guide personalized treatment strategies (Fig. 1) [21]. The synergy between liquid biopsy and AI represents a formidable force in advancing the field of oncology, leading the field towards an era of more precise and data-driven patient care.

Although the field is moving in the right direction, there are evident challenges in liquid biopsy studies, such as the standardization in the pre-analytical phase and sample biobanking (Fig. 1). Pre-analytical factors represent a significant challenge, affecting the reproducibility and accuracy of detection and quantification of biomarkers. Overcoming variability in sample handling (e.g., collection, isolation, short-term storage) and processing is essential for obtaining consistent, reliable, and reproducible results. We therefore highlight the need for amendments to the current Minimum Information for the Publication of Quantitative real-time (MIQE) [22] and digital (dMIQE) [23] PCR Experiments guidelines for analyzing liquid biopsy biomarkers. These amendments would be particularly relevant for liquid biopsy samples for which it may not be possible to create a standard curve or have a pre-established reference gene for relative quantification. Additionally, it is necessary to employ international liquid biopsy pre-analytical standards and coding systems [25] that minimize pre-analytical factors that impact the integrity of liquid biopsy samples during collection, processing, and storage (Fig. 1). These standards could guarantee high-quality samples for research and effective and efficient interconnectivity and interoperability between national and international biobanks.

Establishing and advancing research on liquid biopsies in LMICs is a crucial step toward improving early cancer detection and personalized medicine in regions facing resource constraints. While liquid biopsies offer a non-invasive and cost-effective approach, their implementation in LMICs requires careful consideration of infrastructure, expertise, and financial resources. Initiatives aimed at launching and researching liquid biopsies in LMICs should prioritize capacity building, technology transfer, and knowledge exchange. Collaborations with international institutions can play a pivotal role in providing training for healthcare professionals and scientists, sharing research methodologies, and supporting the development of essential infrastructure. In this context, we advocate for establishing biobanks in LMICs, where research in liquid biopsies is limited, and collaborations with international groups using properly collected, processed, and stored samples might be needed to improve clinical research and eventually launch liquid biopsy-based assays in these regions (Fig. 1).

Finally, the implementation of liquid biopsies into the clinic remains the main goal. To date, numerous liquid-biopsy-based tests have obtained US FDA and European Medicines Agency approval for the analysis of mutations, aberrant expression, or methylation status of solid tumor-associated genes in the clinic. For instance, the Cobas EGFR mutation Test v2 (Roche Diagnostics) was the first approved ctDNA-based companion diagnostic test for non-small cell lung cancer (NSCLC). Additional approved tests include the Epi proColon (blood-based test) and PROGENSA (urine-based test) designed to aid in the early detection of colorectal and prostate cancer through methylation and long non-coding RNA analysis, respectively [24]. Liquid biopsy approaches therefore hold immense promise across various clinical scenarios in precision oncology. From early cancer detection and diagnosis to monitoring treatment response, guiding personalized treatment strategies, and facilitating prognostic assessment, liquid biopsies offer invaluable insights into tumor dynamics and patient outcomes. In addition, the utilization of minimally invasive samples such as urine or saliva as an alternative to blood sampling is gaining increasing attention as they have the potential to reduce the risk of iatrogenic anemia in cancer patients who frequently undergo blood draws. Likewise, these approaches play a critical role in clinical trial enrollment, biomarker validation, and addressing healthcare disparities in LMICs. The development of affordable, rapid, and easy-to-perform tests is a critical aspect of liquid biopsies, underscoring the transformative potential of this technology in revolutionizing cancer care and advancing personalized medicine on a global scale.

Despite the significant advancements made in the development of approved liquid biopsy-based tests, their widespread adoption in the clinic is still facing several challenges. These challenges are primarily attributed to the absence of standardized operating procedures for liquid biopsy analysis in clinical laboratories, as well as regulatory, ethical, and economic hurdles that require careful evaluation. Achieving the full potential of liquid biopsies in routine clinical practice relies on collaborative efforts across scientific, clinical, regulatory, and policy domains to ensure that these innovative techniques improve patient outcomes and quality of life and drive forward the possibilities of personalized medicine in oncology.

## 2 Availability of data and materials

Data sharing does not apply to this article as no datasets were generated or analyzed during the current study.

## Funding

Not applicable.

## Declaration of competing interest

The authors declare that they have no known competing financial interests or personal relationships that could have appeared to influence the work reported in this paper.
